# Distribution, function and evolution characterization of microsatellite in *Sargassum thunbergii* (Fucales, Phaeophyta) transcriptome and their application in marker development

**DOI:** 10.1038/srep18947

**Published:** 2016-01-06

**Authors:** Fuli Liu, Zimin Hu, Wenhui Liu, Jingjing Li, Wenjun Wang, Zhourui Liang, Feijiu Wang, Xiutao Sun

**Affiliations:** 1 Key Laboratory of Sustainable Development of Marine Fisheries, Ministry of Agriculture, Yellow Sea Fisheries Research Institute, Chinese Academy of Fishery Sciences, Qingdao, 266071, China; 2 Laboratory for Marine Fisheries and Aquaculture, Qingdao National Laboratory for Marine Science and Technology, Qingdao, 266237, China; 3 Key Laboratory of Experimental Marine Biology, Institute of Oceanology, Chinese Academy of Sciences, Qingdao, 266071, China; 4 Qinghai Environment Monitoring Centre, Xining, 810007, China

## Abstract

Using transcriptome data to mine microsatellite and develop markers has growingly become prevalent. However, characterizing the possible function of microsatellite is relatively rare. In this study, we explored microsatellites in the transcriptome of the brown alga *Sargassum thunbergii* and characterized the frequencies, distribution, function and evolution, and developed primers to validate these microsatellites. Our results showed that Tri-nucleotide is the most abundant, followed by di- and mono-nucleotide. The length of microsatellite was significantly affected by the repeat motif size. The density of microsatellite in the CDS region is significantly lower than that in the UTR region. The annotation of the transcripts containing microsatellite showed that 573 transcripts have GO terms and can be categorized into 42 groups. Pathways enrichment showed that microsatellites were significantly overrepresented in the genes involved in pathways such as Ubiquitin mediated proteolysis, RNA degradation, Spliceosome, etc. Primers flanking 961 microsatellite loci were designed, and among the 30 pairs of primer selected randomly for availability test, 23 were proved to be efficient. These findings provided new insight into the function and evolution of microsatellite in transcriptome, and the identified microsatellite loci within the annotated gene will be useful for developing functional markers in *S. thunbergii*.

Microsatellites, also known as simple sequence repeats (SSR) or short tandem repeats (STR), are repeated DNA sequences comprising tandem array of short motifs (generally, 1–6 nucleotides), which are widely spread in both eukaryotic and prokaryotic genomes^1^,^2^. Microsatellite was initially used as robust molecular marker and received intensive attention for geneticist and evolutionary biologist. Microsatellite marker is codominant, abundant, multi-allelic, and can be detected by simple and reproducible assays^3^. These important attributes have enabled microsatellite to be powerful marker for molecular population genetics, marker-assisted breeding, DNA fingerprinting, conservation genetics or QTL mapping and positional cloning of target genes^4^,^5^. Despite the wide utilization in the above fields, microsatellite is commonly regarded as “junk” in the genome (i.e. with no significant role as genomic information)^6^, and relative less studies concentrated on its origin, distribution and evolution in genome^1^,^2^,^7^. Recently, as microsatellites have been increasingly identified and characterized within protein-coding genes and their untranslated regions (UTRs), more and more evidences indicate that mi®crosatellite could play a positive role in adaptive evolution via the molecular and phenotypic effects of microsatellite repeat-number variation^6^,^8^.

The main limitation of microsatellite markers development is the requirement of prior characterization of sequences containing microsatellite loci to allow primers design for PCR, making the development of microsatellite markers to be an experimentally long, labor intensive, and costly process^9^. The process involved the construction of genomic libraries using recombinant DNA enriched for a few targeted SSR motifs, and the isolation and sequencing of clones containing microsatellite loci^10^,^11^. With the advent of next-generation sequencing technologies (NGS), it becomes methodologically efficient and economical to sequence the entire genomes or transcriptomes in greater depth than ever before^12^,^13^. Several recent studies have demonstrated that the easiest way to identify microsatellite loci is to mine the genome or transcriptome produced by NGS^9^. The mechanism of microsatellite evolution and the genome-wide distribution were not well understood yet in plants mostly due to the lack of genomic information. The microsatellite identification and characterization in large-scale in genome or transcriptome can thus provide important resource and opportunity to study these themes, together with the development of new genetic markers. In addition, repeat-number variation of microsatellite loci located in or linkage to function genes have impact on chromatin organization, regulation of gene activity, recombination, DNA replication, cell cycle, mismatch repair system^1^,^7^. Previous studies suggest that microsatellites from transcriptome may facilitate us to evaluate the association between microsatellite marker and functional genes or phenotypes^1^.

*Sargassum thunbergii* is an ecologically and economically important brown macroalga. It widely spreads in the intertidal and shallow sublittoral zone along the coasts of China, Japan, and Korea^14^. This species usually grows luxuriantly and can form seaweed forest together with other *Sargassum* spp. and kelp, acting as spawning, nursery and feeding ground for marine animals^15^,^16^. It is good raw material as well for alginate, mannitol, polyphenol and other bioactive substances widely used in chemical, pharmaceutical and food products^17^,^18^,^19^. More recently, *S. thunbergii* has been proposed as a promising candidate alga to construct macroalgal bed in the intertidal zone because of its high tolerance to thermal, osmotic and desiccation stress. In China, the wild resources of *S. thunbergii* were largely eradicated with the rapid growth of holothurian aquaculture industry, as it was regarded as the best natural feed for holothurian. Over the last decade, the technologies for artificial seedling rearing and commercial cultivation of *S. thunbergii* have been developed in China^20^,^21^.

The ecological and economical value of *S. thunbergii* boosted a growing number of studies, especially in the terms of population genetics and genetic improvement, have been conducted to support the development of cultivation industry^22^. To accelerate gene discovery and elucidate the molecular mechanism of special biological processes and ecophysiological characteristics in *S. thunbergii*, we recently sequenced and assembled *de novo* the transcriptome^23^. In the present study, we used the transcriptome data to mine microsatellite loci and characterize their frequency, distribution, and function, and then, we design primers for the amplifying of microsatellite loci and validate the availability of some randomly selected primers. The findings herein will help us to better understand microsatellite evolution in *S. thunbergii* transcriptome, and the developed microsatellite markers can meet the urgent need for studies of population genetics, genetic mapping, and functional gene cloning in *Sargassum* species.

## Results

### Overall characteristics of microsatellite in *S. thunbergii* transcriptome

A total of 46,269 expression sequence tags, obtained from the *de novo* transcriptome sequencing of *S. thunbergii*, were assembled further after redundancy elimination and produced 36,119 consensus sequences with average length of 1,196 bp and N50 of 1,851 bp. These consensus sequences were divided into two groups: 26,451 singletons and 4087 clusters (with over 70% similarities among sequences in clusters) consisting of 9,668 sequences. As showed in the Table 1, a total of 2915 microsatellite loci were identified. Among the 36,119 sequences examined, 2528 (6.70%) harbored microsatellite locus, while only 322 (0.89%) contain more than one microsatellite locus. Most (2822, 96.8%) of the microsatellite loci are the pure or perfect ones, while a small proportion (93, 3.19%) were the compound microsatellites. The frequency or density of microsatellite in *S. thunbergii* transcriptome was 0.068 loci per Kbp.

**Figure 1:**
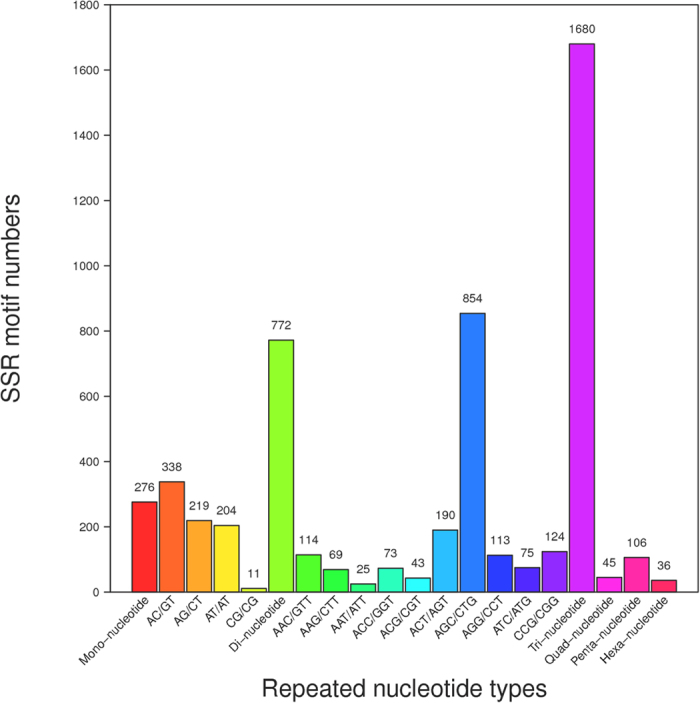
The number distribution of different microsatellite motif types in *Sargassum thunbergii* transcriptome.

Out of the total 2915 SSRs, 1680, 772 and 276 are tri-, di- and mono-nucleotide repeat motif, and 106, 45 and 36 are penta-, tetra- and hexa-nucleotide repeat, respectively. Tri-, di- and mono-nucleotide repeat listed as top three repeat motifs with the largest number, followed by penta-, tetra and hexa-nucleotide (Fig. 1). For the mono-nucleotide motif, G/C was the most abundant type, and the repeat number of mono-nucleotide can reach up to 23 times. For the di-nucleotide motif, AC/GT was the most abundant type with a total of 338 loci, while there was only 11 CG/GC. The length of di-nucleotide tract can reach up to 24 bp implying it can repeat as many as 12 times. There were ten tri-nucleotide motif types, among which AGC/CTG accounted for about 50.8%, while AAT/ATT only accounted for 1.48%. There were 17, 44 and 25 repeat motif types for tetra-, penta and hexa-nucleotide microsatellite, respectively, and the different repeat motif types presented quite evenly.

The average length of the microsatellites was 16.32 bp. The length variation of microsatellite was significantly affected by repeat motif size. Except the length difference between mono- and di-nucleotide as well as tetra- and penta-nucleotide, the length differences among other motif size classes were all statistically significant (*P* < 0.001). The mono-nucleotide has the shortest average length (13.9 bp), while hexa-nucleotide repeat motif was the longest with an average of 25.9 bp. The longest microsatellite identified was 63 bp, with a tri-nucleotide motif repeated 21 times. In addition, the microsatellite length was not significantly affected by base composition (*P* > 0.05).

### Comparison among microsatellites located in CDS, 5′ UTR and 3′ UTR

We investigated the distributional characteristics of microsatellite in the transcript (CDS, 5′ UTR and 3′ UTR). Out of the total 2915 SSRs, 629, 832 and 739 were located in the CDS, 5′ UTR and 3′ UTR, respectively (Table 2). The remaining 715 SSRs were not ascertained because the transcripts containing them lacked enough information to delimit the CDS region. The density or frequency of SSRs in the CDS region is significant lower than that in the UTR region (χ^2^ = 28.16, *P* < 0.01). In other words, the UTR regions harbored more microsatellite compared to the CDS. The motif size classes of microsatellite were significantly affected (χ^2^ = 396.00, *P* < 0.01) by the location (CDS, 5′ UTR and 3′ UTR). For the microsatellite located in the CDS, most of them (91.23%) were the tri-nucleotide. Although the tri-nucleotide microsatellites also dominated in the UTR, the proportions of mono- and di-nucleotide in the UTR were much higher than that in the CDS. The length of microsatellite tract differs significantly between the CDS and the UTR (*P* < 0.01). Compared to the UTR, the microsatellites in the CDS were much shorter; however, the difference in microsatellite length was not statistically significant between 5′ UTR and 3′ UTR (*P* > 0.01).

### Function annotation for genes containing microsatellite

**Figure 2:**
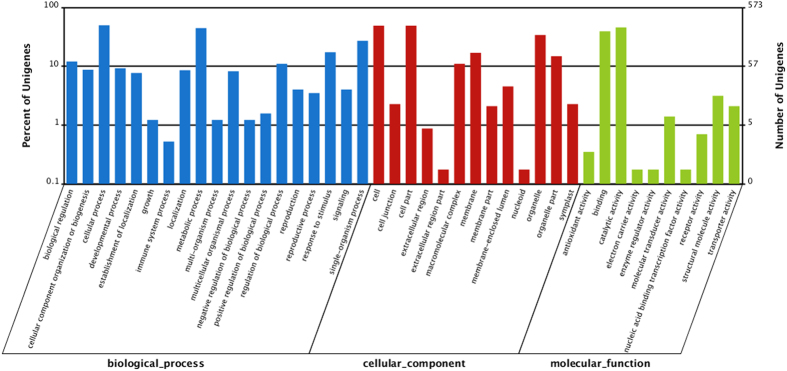
The classification of genes containing microsatellite locus based on the Gene ontology (GO) annotation.

**Figure 3:**
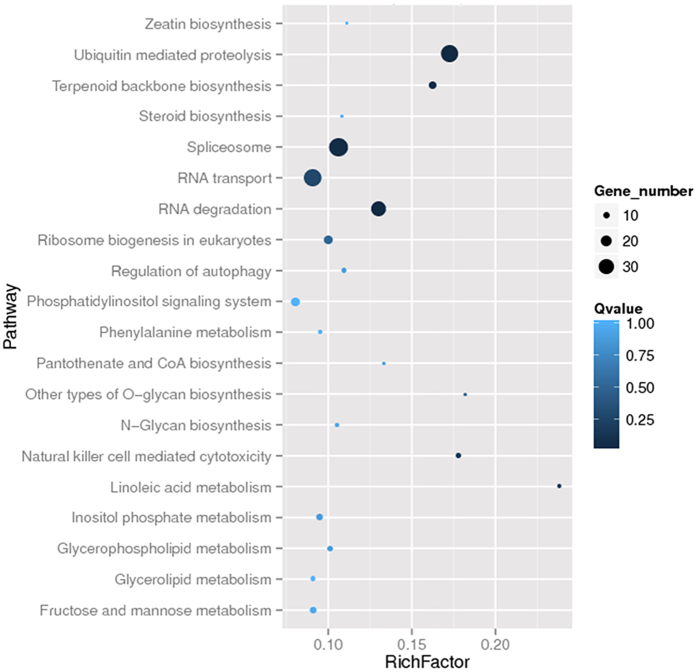
The top 20 enriched pathways involved the gens containing microsatellite locus.

To explore the function of microsatellite, the transcripts containing the SSR was annotated. GO assignment was used to classify the transcripts according to their function. Based on sequence homology, 573 microsatellite-containing transcripts had GO annotations and can be categorized into three functional groups and 42 sub-groups (Fig. 2). For the “biological process” groups, there were 19 subgroups, among which “cellular process”, “metabolic process” and “single-organism process” were the top three sub-groups involved the most genes. For the “cellular component” group, there were 13 subgroups, among which “cell”, “cell part” and “organelle” were the top three sub-groups involved the most genes. Ten sub-groups constitute the “molecular function” group, and the “catalytic activity”, “binding” and “structural molecular activity” involved the most genes. GO enrichment analysis showed that five GO terms (GO:0016747, GO:0004633, GO:0004871, GO:0060089, GO:0042578) over-represented significantly (Q-value < 0.05). The genes involved in the five enriched GO terms possessed the following function respectively: transferring acyl group activity, phosphopantothenoylcysteine decarboxylase activity, signal transducer activity, phosphoric ester hydrolase activity. The function of microsatellite-containing transcripts was further surveyed by the KEGG pathway analysis. The results showed that the transcripts involved in 94 pathways totally. After enrichment analysis, four pathways, that is ko04120 (Ubiquitin mediated proteolysis), ko03018 (RNA degradation), ko03040 (Spliceosome) and ko00900 (Terpenoid backbone biosynthesis), were obtained (Fig. 3).

### Primers design and validation for microsatellite markers

Based on the transcripts containing microsatellite loci, primers flanking 961 microsatellite loci were successfully designed (Table S3). A total of 30 pairs of primer were randomly selected and used to test the availability of these designed primers in a mixed population comprising six individuals. The results showed that seven pairs of primer gave no amplicon, whereas 23 pairs of primer could amplify successfully. Out of the 23 pairs of primer, 21 produced the amplicon with expected size, while 2 gave amplicons larger than the expected size. The 21 pairs of primer were thus used to assess the genetic diversity of a mixed population which consists of six geographic subpopulations with each having five individual. Ten pairs of primer could amplify polymorphic SSR alleles (Table 3). These microsatellite loci in the tested population possessed diverse number of alleles (2~5) with an average of 3.6 (Table 3). Polymorphic Information Content (PIC) of microsatellite markers ranged from 0.339 to 0.694, suggesting that these SSR markers could be used as robust molecular markers for future population genetics, evolutionary analysis or other applications. It is worthy to mention that only three loci (SW9, SW17 and SW18) were in the Hardy-Weinberg equilibrium in the mixed population. Linkage disequilibrium test showed that most of the loci were in linkage equilibrium except three pairs of loci (SW6 and SW17, SW6 and SW18, SW16 and SW35).

## Discussion

In this study, microsatellites in the transcriptome of *S. thunbergii* were mined and characterized. The results showed that microsatellites were only presented in a small proportion of the transcripts (6.70%), consisting with the estimation that 2–11% transcripts contain microsatellite^24^. Recently, more and more ESTs or transcriptome assembled *de novo* were used to mine microsatellite, including several seaweeds such as *Saccharina japonica*^25^,^26^, *Laminaria digitata*^27^, *Pyropia*^28^, etc. Although the frequency or density of microsatellite differs slightly in different species due to the varied criteria used in microsatellite identification, it demonstrated that the transcriptome is an invaluable resource for microsatellite identification. These identified microsatellite loci can promote the molecular maker development in *S. thunbergii* and have potential application in other Sargassaceae species.

Our results showed that microsatellite loci in *S. thunbergii* transcriptome do not distribute evenly in UTR and CDS regions, with more prevalence in the former than in the latter. The relative prevalence of microsatellites in UTRs was consistent with the results of previous transcriptome surveys^29^,^30^,^31^,^32^. The density of microsatellite in genome may be depended on or affected by two factors: 1) evolutionary constrains on microsatellite due to the harmful effect of microsatellite mutation on gene function, and 2) the direction selection on microsatellites with adaptive roles^33^,^34^,^35^. As microsatellites are highly prone to “indel” mutations by means of slip-strand mispairing^33^, microsatellites in the CDS region are more likely to damage normal gene function than microsatellite in the UTR regions, leading to higher evolutionary constrains on microsatellites in the CDS region. Conversely, the URT regions exhibited higher tolerance for mutation, and possessed higher prevalence of microsatellite due to the lower evolutionary constrains. Moreover, microsatellites in the UTRs may have the “tuning ability” on functional genes^34^,^35^, conferring the genes adaptive roles in evolution. Thus, the directional selection on these microsatellites probably favored microsatellite expansion in UTRs.

We found that the Tri-nucleotide was the most abundant among the six motif size classes, consistent with the studies in plants such as *Arabidopsis thaliana* and rice^29^, *Brassica rapa*^31^, *Medicago tunculata*^30^ and *Helianthus annuus*^32^. The higher prevalence of Tri-nucleotide may be due to that this type of microsatellites should be less likely to cause frameshift mutations^36^. The length of microsatellite tract also reflects the effect of evolution and selection on microsatellite loci development. Our study found that the length variation of microsatellite tract was significantly affected by both the repeat motif size classes and the location (in CDS or UTR). Compared to other motif size classes, tri- and hexa-nucleotide were much longer, suggesting the lower evolution constrains on tri- and hexa-nucleotide because these two type microsatellite do not cause frameshift mutation in genes. Moreover, microsatellites in UTR were much longer than those in CDS, reflecting higher evolution constrains on the microsatellites in CDS than in UTR regions.

SSRs are previously regarded as ‘junk’ in genome or as evolutionarily neutral DNA markers. However, microsatellites have growingly been found and characterized within protein-coding genes and their untranslated regions, providing multiple lines of evidence for the function and evolution of microsatellite^7^,^8^. When microsatellites locate in or link to functional gene, their repeat motif variation, which is frequently and reversibly by adding or subtracting motif, will influence on gene regulation, transcription, translation and protein function^6^. In this study, we found that microsatellite were over-represented in genes involved in pathways such as Ubiquitin mediated proteolysis, RNA degradation, Spliceosome and Terpenoid backbone biosynthesis (Fig. 3). The ubiquitin proteolytic system plays important roles in a broad array of basic cellular processes by selective proteolysis and in plant response and adaptation to drought, salinity, cold and nutrient deprivation^37^. *S. thunbergii* inhabits in the intertidal zone and frequently suffers severe adverse conditions such as thermal, osmotic, illumination, and desiccation stresses as tides rise and fall^38^. Previous study showed that *S. thunbergii* possesses innate tolerance to these stresses^16^, allowing us to postulate that microsatellite located in genes related to Ubiquitin mediated proteolysis may be ecologically crucial for *S. thunbergii* to accumulate adaptive genetic variation to adapt to harsh environment variables. Microsatellite was also proved to be related to stress adaptation in other organisms. For example, the microsatellites identified in *Bemisia tabaci* transcriptome were located in the genes related to resistance to environmental stresses and insecticides such as aldehyde oxidase, cytochrome P450 and mitogen-stress activated protein kinases^39^. The significant enrichment of microsatellite in *Helianthus annuus* transcriptome was observed in GO terms associated with biological processes that involved in plant response to stress, biotic and abiotic stimuli^32^.

We also found that partial transcripts harboring microsatellite have the transcription factor activity. A similar investigation of microsatellite in the genome of rice and *Arabidopsis* showed that some transcripts harboring microsatellite were also related to the transcription factors^29^. In *Elaeis guineensis*, microsatellite polymorphisms were found in sequences encoding AP2-like, bZIP, zinc finger, MADS-box, and NAC-like transcription factors^40^. Transcription factors, as the crucial factors in transcription regulation, play a crucial role in plant growth, development and evolution^41^. Microsatellite in transcription factor coding gene may function as important “Tuning Knob” in evolution^34^,^35^. Moreover, microsatellites were also overrepresented in other pathways (Fig. 3), such as “RNA transport”, “Ribosome biogenesis in eukaryotes”, “regulation of autophagy”, although they were not significantly enriched (Q-value > 0.05). In brief, the microsatellite loci and their association to gene function or pathways may shed light on the function of microsatellite. However, because of the limitation of the hypergeometric test used here, it must be careful to make a final conclusion about the function of microsatellite loci. It needs more genetic experiments to validate the probable function annotated by bioinformatics method.

Numerous studies have demonstrated that the EST or transcriptome produced by NGS was valuable resources to efficiently develop SSR markers in large-scale^9^. Herein, we identified applicable microsatellite loci and some of them have been proved as efficient molecular markers. To the best of our knowledge, these are the first set of microsatellites identified in *S. thunbergii*. Given the high transferability of genic- or EST-SSR markers, the microsatellites identified from *S. thunbergii* will have wide application in other *Sargassum* species with limited number of SSR markers^42^,^43^. Previous study revealed low to moderate levels of genetic variations (*H*_*E*_: average expected heterozygosity, varying from 0.2729 ~ 0.2903) within *S. thunbergii* population using random amplified polymorphic DNA (RAPD) and inter-simple sequence repeat (ISSR) markers^22^. With the ten microsatellite markers, we found the genetic diversity of *S. thunbergii* population was much higher (*H*_*E*_: 0.422-0.754). Microsatellite markers can generally reveal higher genetic diversity than RAPD markers^3^. However, one noteworthy factor here is that the tested population was a mixed population consisting of six geographic subpopulations (from Liaoning, Shandong and Zhejiang Province, China) with each having five individuals, whereas the populations in the previous study were the local populations in Shandong Province^22^.

## Materials and Methods

### Microsatellite identification and characterization

A total of 46,269 expression sequence tags, obtained from the *de novo* transcriptome sequencing of *Sargassum thunbergii*^23^, were further assembled with Phrap (http://www.phrap.org/) after redundancy elimination by TIGR v2.1^44^. These assembled consensus sequences were used to identify microsatellite loci using software MISA (Microsatellite searching tool, http://pgrc.ipk-gatersleben.de/misa/) with the following criteria: mono-nucleotide repeats motif with at least 12 repeats, di-nucleotide with six, tri- and quad-nucleotide with five, penta and hexa-nucleotide with four. The criterion for compound microsatellites is that the interval between two repeat motifs was shorter than 100 nt.

In order to investigate the distribution of microsatellite in *S. thunbergii* transcriptome, the relative position of microsatellites with regard to start and stop codons was inferred to determine whether microsatellites were in 5′UTR, CDS or 3′UTR. The position of start and stop codon in sequences were inferred by comparing potentially homologous sequences in Swissprot database, using the software package ESTScan ver. 2.0^45^,^46^. The location of microsatellite was determined based on the predicted CDS, 5′ UTR and 3′ UTR region. In order to understand the evolution of microsatellites in *S. thunbergii* transcriptome, several characteristics of microsatellite, such as, prevalence or density (one loci per Kbp), motif size (motif length) , motif type (base composition), were determined and compared with each other among microsatellite loci located in CDS, 5′ UTR and 3′ UTR. Chi-square analyses were conducted to test whether the density of microsatellite in different transcript domains (CDS, 5′ UTR and 3′ UTR) is significant according to the previously reported method^32^. Kruskal–Wallis rank sum test^47^ was conducted to test whether the microsatellite length was affected by (i) transcript domains, (ii) motif size, and (iii) motif type. The detailed analysis method and process followed the previous report^32^.

### Function annotation of genes containing SSR

To understand the possible function of microsatellite, all the transcripts harboring microsatellite were searched against the GenBank nr protein database using BLASTx with an E-value cut-off of 10^−5^. Blast2GO program^48^ was used to get GO annotation, and WEGO software^49^ to classify the transcripts. To investigate whether some GOs or pathways exhibited microsatellite enrichment, hypergeometric tests were used to determine whether microsatellite-containing transcripts ascribed to specific GOs or pathways are more likely to encode microsatellites than expected by chance. The calculating formula for the P-value is as follows:


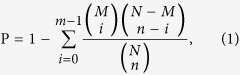


where N is the number of all transcripts that with GO or KEGG annotation, n is the number of transcripts containing microsatellite in N, M is the number of all genes annotated to specific GO or pathways, and m is number of transcripts containing microsatellite in M. Q-value was obtained from P-value by Bonferroni correction. GOEAST^50^ was used to carry out GO enrichment analysis, identifying the overrepresented GO terms. For pathway enrichment analysis, all transcripts harboring microsatellite were assigned to terms in KEGG database^51^ and searched for significantly enriched KEGG terms compared to the whole transcriptome background. The statistical analysis was accomplished by a customized Perl script with the same formula mentioned above.

### Primer design and validation for microsatellite loci

To explore the diversity and mutability of the microsatellite loci identified in the *S. thunbergii* transcriptome, the primers were designed based on the sequences flanking the microsatellite loci using Primer3-2.3.4 with default parameters. A total of 30 pairs of primer were selected randomly and used to amplify the microsatellite loci in a mixed population comprising six geographic subpopulations with each having five individuals (Table S1). Genomic DNA was extracted using a Pant genomic DNA kit (Tiangen Biotech CO., Ltd, Beijing, China) according to the manufacturer’s instructions. Polymerase chain reactions (PCRs) were carried out in a total volume of 20 μL containing 0.5 U Taq DNA polymerase (MBI), 1 × PCR buffer, 0.2 mM dNTP mix, 0.5 μM of each primer set, 2.0 mM MgCl_2_ and about 50 ng template DNA. The mixture was subjected to 94 °C for 4 min, following by 35 cycles of 30 min at 94 °C, 30 s at annealing temperatures (refer to Table 2), 40 s min at 72 °C, and a final step at 72 °C for 10 min. PCR products were resolved via 6% denaturing polyacrylamide gel, and visualized by silver-staining^52^. The observed number of alleles (Na), the mean observed heterozygosity (H_O_) and the mean expected heterozygosity (H_E_) for each locus in the tested population were calculated by genetic analysis package POPGENE version 1.3^53^. Tests of the Hardy-Weinberg equilibrium and linkage disequilibrium for these loci in the test population were performed using GENEPOP^54^.

## Additional Information

**How to cite this article**: Liu, F. *et al.* Distribution, function and evolution characterization of microsatellite in *Sargassum thunbergii* (Fucales, Phaeophyta) transcriptome and their application in marker development. *Sci. Rep.* **6**, 18947; doi: 10.1038/srep18947 (2016).

## Supplementary Material

Supplementary Table S1

Supplementary Table S2

## Figures and Tables

**Table 1:** General information for microsatellite analysis.

Items	Number
Total number of sequences examined	36119
Total size of examined sequences (bp)	43180505
Total number of identified SSRs	2915
Number of sequence containing SSR	2528
Number of sequences containing more than one SSR	322
Number of SSRs present in compound formation	93
Number of SSR per kbp	0.068

**Table 2:** Distribution and Characteristics of microsatellites in different transcript regions.

Region	Total number of base pair (bp)	Number of SSR	Number of SSR per kpb	Mean length of SSR (bp)
Coding	761730	629	0.83	16.35
UTR	1753943	1571	0.90	16.49
5′UTR	892254	832	0.93	16.53
3′UTR	861689	739	0.86	16.44

**Table 3:** Microsatellite markers development and their application in a tested population.

Loci	Primer sequences (5′-3′)	Repeat motif	Ta (°C)	Size range (bp)	N_A_	PIC	H_O_	H_E_
SW1	F:AACGGAAGCGCAATACGAC	(AC)_11_	60	411–421	5	0.653	0.633	0.720
	GCAGACACGGTTGACGAAG							
SW6	CAAAGTTGCTGCGTGATTCG	(CT)_11_	60	160–164	2	0.375	0.433	0.508
	CACGATGTGTCGCCTTCTG							
SW9	AAAGTTGCTGAGCCGTTCG	(ACC)_8_	60	435–456	4	0.694	0.433	0.754
	CAGGAGGACCATCGATCCC							
SW10	TGGCTGTGTGGATACGACC	(ACT)_8_	60	332–353	5	0.651	0.566	0.714
	TGTCGCAATGCTCGTTGTAG							
SW16	CCCAAATCAGCGAAAGGCG	(GTT)_21_	61	402–411	3	0.342	0.333	0.422
	CGGTGCTACGATACTGCCC							
SW17	GCCTTCGTTACGCTTGACC	(ATTT)_6_	60	364–372	5	0.420	0.100	0.473
	TACCACCTGAGCAATCCCG							
SW18	ACCCGACGAGCTCTACAAG	(CAGT)_6_	60	428–452	5	0.427	0.333	0.465
	TGAGTGGGTTGAAGACGGG							
SW21	ATGCCAGGAGCTACACAGG	(AAACC)_7_	60	383–400	4	0.418	0.600	0.532
	AGATGGCTCAACCTCTGCC							
SW24	TTGCCCGGGTATCCTGTTC	(AAACAT)_4_	60	310–316	2	0.339	0.300	0.440
	TTTCGCGTTGAGCACTTCG							
SW35	GCTATGTCAACAACCACCTCT	(ATC)_4_…(ATC)_4_	59	332–348	4	0.411	0.500	0.521
	TTCTGATTCGAGGTATTGTGC							

Ta: annealing temperature; N_A_: The observed number of alleles; PIC: Polymorphism Information Content; Ho: the mean observed heterozygosity; H_E_: the mean expected heterozygosity.
