# Supplementary material for: Distribution, function and evolution characterization of microsatellite in Sargassum thunbergii (Fucales, Phaeophyta) transcriptome and their application in marker development
Source: Sci Rep. 2016 Jan 6;6:18947. doi: 10.1038/srep18947 (PMC4702172; doi:10.1038/srep18947)
Supplement: Supplementary Table S1 [file srep18947-s1.pdf]

Distribution, function and evolution characterization of microsatellite in *Sargassum thunbergii* (Fucales, Phaeophyta) transcriptome and their application in marker development

Fuli Liu, Zimin Hu, Wenhui Liu, Jingjing Li, Wenjun Wang, Zhourui Liang, Feijiu Wang, Xiutao Sun

Table S1: the detailed information for the Sargassum populations.

Population code	Number of individual	Location	Longitude and latitude	Sampling time
ZZ	5	Zhangzidao	39.01 °N, 122.73 °E	2014/7/12
JM	5	Jimingdao	37.75 °N, 122.80 °E	2014/6/26
YL	5	Yuliangwan	37.51 °N, 121.43 °E	2014/3/22
CS	5	Chengsantou	37.39 °N, 122.71 °E	2014/6/14
NJ	5	Nanjidao	27.45 °N, 121.05 °E	2014/4/17
DT	5	Dongtou	27.80 °N, 121.14 °E	2010/6/6
